# The Salivary IgA Flow Rate Is Increased by High Concentrations of Short-Chain Fatty Acids in the Cecum of Rats Ingesting Fructooligosaccharides

**DOI:** 10.3390/nu8080500

**Published:** 2016-08-17

**Authors:** Yuko Yamamoto, Toru Takahahi, Masahiro To, Yusuke Nakagawa, Takashi Hayashi, Tomoko Shimizu, Yohei Kamata, Juri Saruta, Keiichi Tsukinoki

**Affiliations:** 1 School of Dental Hygiene, Department of Junior College, Kanagawa Dental University, Yokosuka, Kanagawa 238-8580, Japan; yamamoto.yuko@kdu.ac.jp; 2 Department of Nutrition and Health Sciences, Fukuoka Women’s University, Fukuoka 813-8529, Japan; takahashi@fwu.ac.jp; 3 Division of Environmental Pathology, Department of Oral Science, Kanagawa Dental University, Graduate School of Dentistry, Yokosuka, Kanagawa 238-8580, Japan; m.tou@kdu.ac.jp (M.T.); yusuke_2828@yahoo.co.jp (Y.N.); hayashi-t@plum.plala.or.jp (T.H.); saruta@kdu.ac.jp (J.S.); 4 Department of Highly Advanced Stomatology, Kanagawa Dental University, Graduate School of Dentistry, Yokohama, Kanagawa 221-0835, Japan; shimizu@kdu.ac.jp (T.S.); kamata@kdu.ac.jp (Y.K.)

**Keywords:** IgA, saliva, short-chain fatty acid, fructooligosaccharides, rats

## Abstract

Salivary immunoglobulin A (IgA) serves as a major effector in mucosal immunity by preventing submucosal invasion of pathogens. However, the mechanism by which consumption of fermentable fibers increases IgA in saliva was not fully elucidated. This study investigated the effects of fructooligosaccharides (FOS) intake and time after feeding on IgA levels in the saliva and cecal digesta and on the concentration of short-chain fatty acids (SCFA) in the cecum in rats. Five-week-old rats were fed a fiber-free diet or a diet with 50 g/kg FOS for zero, one, four, and eight weeks. Ingestion of FOS at one and eight weeks led to a higher IgA flow rate of saliva per weight of submandibular gland tissue (*p* < 0.05), which positively correlated with the concentration of SCFA in the cecal digesta (*r*_s_ = 0.86, *p* = 0.0006, *n* = 12), but showed no correlation with the concentration of IgA in the cecal digesta (*r*_s_ = 0.15, *p* = 0.3, *n* = 48). These results suggested that ingestion of FOS increased salivary IgA secretion through high levels of SCFA in the large intestine, which was produced by fermentation of FOS. Thus, continuously ingesting FOS for more than one week could increase secretion of salivary IgA.

## 1. Introduction

The oral cavity is the beginning of the aerodigestive tract [1] and is, thus, constantly exposed to microorganisms that could colonize and lead to disease [2]. To protect against pathogen invasion, the oral cavity is covered by the mucosal epithelium, which houses the mucosal immune system [1]. The oral cavity is one of the effector organs in the mucosal immune system and closely related to the intestinal tract [3]. This region is constantly bathed in the saliva secreted by major and minor salivary glands [4], which provides a continuous rich source of electrolytes, mucus, antibacterial compounds, and enzymes that support lubrication and initiate food digestion [5]. Saliva includes many kinds of antibacterial materials [6], including immunoglobulin A (IgA), a major effector in mucosal immunity that prevents submucosal invasion of pathogens. When pathogens enter through the oral cavity, the antiviral and antibacterial effects of IgA prevent viral replication and bacterial attachment to the mucosal surfaces [7] by specifically binding to the pathogens [3]. The salivary glands are the most important IgA-producing organ in the upper respiratory tract [8]. Low levels of IgA in saliva are associated with a high incidence and recurrence of upper respiratory tract infections (URTI) [7,9,10]. Children with no detectable IgA in their serum or whole saliva frequently develop bronchopulmonary infections, bronchial asthma, gastrointestinal allergy, and other conditions [11]. Thus, salivary IgA plays very important roles in protecting the body and oral cavity against infectious diseases. Since salivary IgA is generally present at lower levels in children and the elderly than in adults [12,13], maintaining high salivary levels of IgA is particularly important for URTI prevention in young and elderly populations.

Ingestion of fermentable fibers by the intestinal microbiota could mediate immune changes via direct contact between lactic acid bacteria or bacterial products and immune cells in the intestine, the production of short-chain fatty acids (SCFA) from fiber fermentation, or changes in mucin production [14]. Fructooligosaccharides (FOS) reaches the large intestine where they increase SCFA levels [15]. FOS consumption leads to high levels of IgA in digesta in the large intestine and feces in humans and animals [15,16,17]. IgA in the large intestine also prevents mucosally-transmitted pathogens and commensal bacteria from binding to epithelial cells and neutralizes their toxins to maintain homeostasis [18].

Although many reports have indicated that IgA increases in the large intestine after FOS intake [19], the influence of the FOS intake on salivary IgA remains unclear. Our previous study indicated that ingestion of FOS and a mixture of polydextrose and lactitol for three weeks increased IgA levels in both the large intestine and the saliva of rats and was the first to demonstrate the interrelationship between ingestion of indigestible carbohydrates and salivary IgA levels [20]. However, the mechanism by which IgA is increased in saliva by consumption of fermentable fibers, such as FOS, was not fully elucidated. Therefore, the purpose of the present study was to examine the effect of FOS intake and time after feeding on IgA levels in the saliva and cecal digesta and on fermentation of FOS in the cecum of rats.

## 2. Materials and Methods

### 2.1. Animals

The experimental protocol used in this study was reviewed and approved by the Ethics Committee for Animal Experiments of Kanagawa Dental University (approval number 20140724) and was performed in accordance with the Guidelines for Animal Experimentation of Kanagawa Dental University and the Animal Research: Reporting of In Vivo Experiments (ARRIVE) guidelines for reporting animal research. Four-week-old male Wistar rats were purchased from CLEA Japan (Tokyo, Japan) and housed in wire mesh cages without bedding material at 22 ± 3 °C with a 12 h light/12 h dark cycle. The rats had free access to a commercial diet (CE-2, Japan CLEA) and water for seven days before starting the experiment. Thereafter, we randomly divided the 48 rats into eight groups (see Diets and Sampling) maintained under equivalent conditions except diet composition and experiment duration; no rats died during the experiment. The rats had free access to diet and water. All rats were sacrificed by decapitation between 7:00 and 11:00 after anesthetization with pentobarbital and collection of blood samples by cardiac puncture.

### 2.2. Diets

The compositions of the fiber-free control diet and experimental diet are shown in Table 1. The control diet was based on the solidified AIN-76 formulation with all corn starch and cellulose converted to sucrose. FOS (Meioligo-P^®^, Meiji Food Material Co. Ltd., Tokyo, Japan) was added at 50 g/kg for the FOS diet, which was prepared by Japan CLEA. FOS consisted of 340 g/kg 1-kestose, 530 g/kg nystose and 100 g/kg fructofuranosyl nystose. We employed AIN-76 to eliminate the flux of carbohydrates, such as corn starch and cellulose, which also produce SCFA [21,22], to the large intestine. Sucrose is absorbed in the small intestine and does not reach the large intestine; thus, sucrose does not influence fermentation. Consumption of AIN-76 results in lower levels of SCFA in the feces of rats compared with consumption of AIN-93, which is based on corn starch [23]. Thus, the FOS diet used in our study reduces the influence of SCFAs generated from corn starch, thereby allowing the influence of FOS fermentation to be determined. On average, a 30 g diet was fed to each rat daily.

### 2.3. Sampling

After zero, one, four, or eight weeks, all rats were anesthetized with sodium pentobarbital (65 mg/kg body weight; Kyoritsu Seiyaku Corporation, Tokyo, Japan) for sampling. Saliva was collected under anesthesia. After sacrifice, the cecal digesta, cecal tissues, and submandibular glands were excised. All samples were collected between 7:00 and 11:00 and weighed immediately, then stored at −20 °C until analysis. 

### 2.4. Collection of Saliva

Five minutes after anesthetization with sodium pentobarbital, salivary secretion was elicited by intraperitoneal injection of pilocarpine (8 mg/kg body weight, NACALAI TESQUE, INC., Kyoto, Japan). After salivation began (~5 min after injection), whole saliva was collected by pipette for 10 min. All saliva samples were stored at −20 °C until analysis.

### 2.5. Measurement of IgA Concentration

The concentration of IgA in the cecal digesta and saliva was quantified by ELISA using a Rat IgA ELISA Quantitation Kit (Bethyl Laboratories, Montgomery, TX, USA). Cecal digesta samples were treated with ten volumes of distilled water with 1 mM phenylmethylsulfonyl fluoride (PMSF) from dimethyl sulfoxide (DMSO)-dissolved stock for 60 min at room temperature. The samples were then centrifuged (10,000× *g*, 15 min, 4 °C), and the supernatant fractions were used for IgA measurement.

Ninety-six-well microtiter plates were coated for 1 h at room temperature with goat anti-rat IgA (1:100 dilution) diluted with the coating buffer (0.05 M carbonate-bicarbonate, pH 9.6). The residual fluid was decanted and wells washed five times with wash solution (50 mM Tris, 0.14 M NaCl, 50 g/L Tween 20, pH 8.0). Blocking solution (50 mM Tris, 0.14 M NaCl, 10 g/L bovine serum albumin, pH 8.0) was added to each well and the plate incubated for 30 min at room temperature before washing with wash solution. Samples and rat IgA standards (Bethyl Laboratories) were added to respective wells. The plate was incubated for 1 h at room temperature and washed five times with wash solution. Horseradish peroxidase (HRP)-conjugated goat anti-rat IgA antibody (1:15,000 dilution) was added to each well and the plate was incubated for 1 h at room temperature. The enzyme substrate 3,3′,5,5′-tetramethylbenzidine (TMB) peroxidase solution was added to each well after washing. The plate was developed in the dark at room temperature for 15 min. The reaction was stopped with 0.18 M H_2_SO_4_ stop solution. Absorbance was measured at 450 nm in an automated microplate reader (BioRad, Hercules, CA, USA).

The flow rate of saliva (mL/min) was divided by the weight of saliva (mg) with the sampling time (10 min) assuming that the specific density of saliva was 1.00 g/mL. The IgA flow rate of saliva (μg/min) was calculated by multiplying the absolute concentration of IgA (μg/mL) by the saliva flow rate (mL/min). In the present study, the IgA flow rate of saliva positively correlated with the weight of submandibular gland tissue (*r*_s_ = 0.78, *p* < 0.0001, *n* = 48, Spearman’s rank correlation coefficients), suggesting the weight of submandibular gland tissue affected the IgA flow rate of saliva. Accordingly, the IgA flow rate of saliva was divided by the weight of the submandibular gland tissue to remove this influence. The weight of submandibular gland tissue was calculated as the median weight of the right and left tissue.

### 2.6. Measurement of pH in Cecal Digesta

The pH of cecal digesta was measured with a compact pH-measuring instrument (HORIBA, Ltd., Kyoto, Japan). All cecal digesta samples were treated with equal volumes of distilled water. After thorough mixing, samples were centrifuged (10,000× *g*, 15 min, 4 °C), and the supernatant fractions were used for pH measurement.

### 2.7. Measurement of Organic Acids in Cecal Digesta

Preprocessing of cecal digesta and analysis of organic acid concentrations were based on the methods of Tsukahara et al. [24]. Samples (0.3 g) were mixed with 0.6 mL distilled water. Diluents were mixed with 90 μL 120 g/L perchloric acid After centrifugation (15,000× *g*, 10 min, 4 °C), the supernatant fractions were filtered through a 0.45-μm cellulose acetate membrane filter (Cosmonice Filter W, Nakalai Tesque, Kyoto, Japan) and degassed under vacuum. The supernatant fractions (5 μL) were injected into an SIL-10 autoinjector (Shimadzu, Kyoto, Japan). Organic acids were separated by two serial organic acid columns (Shim-pack SCR-102H, Shimadzu, Kyoto, Japan) with a guard column (SCR-102HG; Shimadzu, Kyoto, Japan) at 45 °C with isocratic elution (0.8 mL/min) of 5 mM *p*-toluenesulfonic acid aqueous solution using a solvent delivery pump (LC-10ADvp; Shimadzu, Kyoto, Japan) with an online degasser (DGU-12A; Shimadzu, Kyoto, Japan). Organic acids were detected with an electronic conductivity detector (Waters 431; Waters Corporation, Milford, MA, USA) after post-column dissociation (0.8 mL/min) with 5 mM *p*-toluenesulfonic acid, 20 mM bis-Tris, and 100 μM ethylenediaminetetraacetic acid by using the solvent delivery pump. Organic acids were quantified with a system controller (CBM-20A; Shimadzu, Kyoto, Japan).

### 2.8. Bayesian Network

A Bayesian network is a directed acyclic graph that is composed of a set of variables {*X*_1_, *X*_2_, ... , *X_N_*} and a set of directed edges between the variables [25]. Bayesian networks are very successful in probabilistic knowledge representation and reasoning. In Bayesian networks, the joint probability distribution function of all nodes can be calculated as follows:

(1) $$P\;(X_{1},X_{2},\;\ldots ,\;X_{N})\;=\;\prod_{i=1}^{N}P\;(Xi|\;Pai)$$

where *Pa_i_* is the set of random variables whose corresponding nodes are parent nodes of *X_i_*.

A Bayesian network contains two elements: structure and parameters. Each arc begins at a parent node and ends at a child node. *Pa* (*X*) represents the parent nodes of node *X*. *X*_1_ is the root node because it has no input arcs. Root nodes have prior probabilities. Each child node has conditional probabilities based on the combination of states of its parent nodes.

### 2.9. Statistical Analysis

All statistical analyses were performed using JMP version 12 (SAS Institute Japan, Tokyo, Japan) and R version 3.2.0 (The R Project for Statistical Computing, Vienna, Austria, 2013). Results were expressed as the mean and standard error (SE). Statistical analyses of time-series data were performed using factorial two-way ANOVA which showed two main effects and an interaction effect. Tukey’s multiple comparison for interaction was used for post hoc analysis when the interaction was significant. Spearman’s rank correlation was employed to detect correlation between two variables. Welch’s *t*-test was used to compare the concentration of organic acids and SCFA in cecal digesta from the FOS and control groups. Causal effects between variables were calculated using Bayesian network analysis. *P*-values less than 0.05 were considered statistically significant.

## 3. Results

### 3.1. Effects of Addition of FOS and Times after Feeding

**Figure 1:**
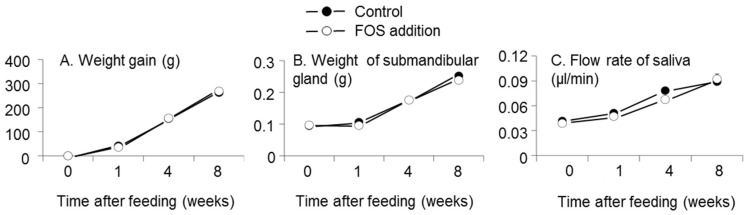
Effects of fructooligosaccharides (FOS) addition and time after feeding on the weight gain (**A**); flow rate of saliva (**B**); and weight of submandibular gland (**C**). *n* = 6 per group at each time. Symbols and vertical bars represent means and standard errors, respectively. There were no effects of interaction and FOS addition on weight gain, flow rate of saliva, and weight of submandibular gland (interaction: *p* = 0.8, 0.5, and 0.7, respectively; FOS addition: *p* = 0.9, 0.2, and 0.4, respectively, two-way analysis of variance (ANOVA)). Time after feeding affected weight gain, flow rate of saliva, and weight of submandibular gland (*p* < 0.0001, *p* < 0.0001, *p* < 0.0001, respectively).

There were no effects of interaction between FOS supplementation and time after feeding or of FOS supplementation on weight gain, flow rate of saliva, or weight of submandibular gland tissue (interaction: *p* = 0.8, 0.5, and 0.7, respectively; FOS addition: *p* = 0.9, 0.2, and 0.4, respectively, two-way analysis of variance (ANOVA), Figure 1). Time after feeding affected weight gain, flow rate of saliva, and weight of submandibular gland tissue (*p* < 0.0001, *p* < 0.0001, *p* < 0.0001, respectively, two-way ANOVA).

**Figure 2:**
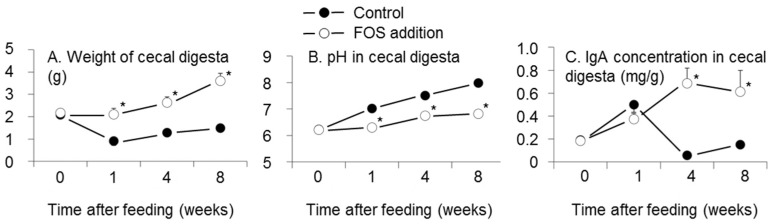
Effects of fructooligosaccharides (FOS) addition and duration of feeding for test diets on the weight of cecal digesta (**A**); pH in cecal digesta (**B**); and IgA concentration in cecal digesta (**C**). *n* = 6 per group at each time. Symbols and vertical bars represent means and standard errors, respectively. There were interactions between FOS addition and intake period in the weight of cecal contents, pH in cecal digesta, and IgA concentration in cecal digesta (*p* = 0.0002, *p* < 0.0001, and *p* < 0.0001, respectively, two way ANOVA). * *p* < 0.05 versus control group at each week using Tukey’s multiple comparison.

Interactions between FOS addition and time after feeding existed for the weight of cecal digesta, pH in cecal digesta, and IgA concentration in cecal digesta (*p* = 0.0002, *p* < 0.0001, and *p* < 0.0001, respectively, two-way ANOVA, Figure 2). The weight of cecal digesta in the FOS group was higher than that of the control group at one, four, and eight weeks (*p* < 0.05, Tukey’s multiple comparison for interaction, Figure 2A). The pH in cecal digesta in the FOS group was lower than that of the control group at one, four, and eight weeks (*p* < 0.05, Figure 2B). The IgA concentrations in cecal digesta at four and eight weeks were higher than that at week zero in the FOS group (*p* < 0.05, Figure 2C). 

**Figure 3:**
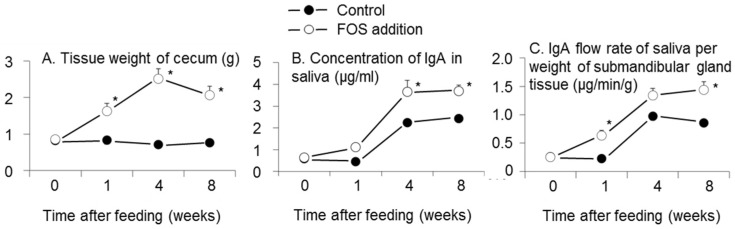
Effects of fructooligosaccharides (FOS) addition and time after feeding on the tissue weight of cecum (**A**); concentration of IgA in saliva (**B**); and IgA flow rate in saliva per weight of submandibular gland tissue (**C**). *n* = 6 per group at each time. Symbols and vertical bars represent means and standard errors, respectively. There were interactions between FOS addition and intake period in the weight of cecal tissue, concentration of IgA, and IgA flow rate of saliva per weight of submandibular gland tissue (*p* < 0.0001, 0.03, and 0.0009, respectively, two-way ANOVA). * *p* < 0.05 versus control group at each week using Tukey’s multiple comparison.

Interactions between FOS addition and time after feeding were observed in the weight of cecal tissue, concentration of IgA in saliva, and IgA flow rate of saliva per weight of submandibular gland tissue (*p* < 0.0001, 0.03, 0.0009, respectively, two-way ANOVA, Figure 3). The weight of cecal tissue in the FOS group was higher than that of the control group at one, four, and eight weeks (*p* < 0.05, Tukey’s multiple comparison for interaction, Figure 3A). In the FOS group, the weight of cecal tissue at one, four, and eight weeks was higher than that at week zero (*p* < 0.05, Figure 3A). The concentration of IgA in saliva in the FOS group was higher than that of the control group at four and eight weeks (*p* < 0.05, Figure 3B). The concentration of IgA in saliva at four and eight weeks was higher than that at week zero in both of FOS and control groups (*p* < 0.05, Figure 3B). The IgA flow rate of saliva per weight of submandibular gland tissue in the FOS group was higher than that of the control group at one and eight weeks (*p* < 0.05, Tukey’s multiple comparison for interaction, Figure 3C). In the FOS group, the IgA flow rate of saliva per weight of submandibular gland tissue at one, four, and eight weeks was higher than that at week zero (*p* < 0.05). In the control group, the IgA flow rates of saliva per weight of submandibular gland tissue at four and eight weeks were higher than that at week zero (*p* < 0.05, Figure 3C). 

### 3.2. Effect of FOS on Concentration of SCFA in Cecal Digesta

The concentrations of *n*-butyrate and total SCFA in cecal digesta in the FOS group were higher than those in the control group (*p* = 0.004, *p* < 0.05, respectively, Welch’s *t*-test, Table 2). 

### 3.3. Relationship with IgA Flow Rate of Saliva per Weight of Submandibular Gland Tissue

The IgA flow rate of saliva per weight of submandibular gland tissue positively correlated with the concentration of SCFA in cecal digesta (*r*_s_ = 0.86, *p* = 0.0006, *n* = 12), the weight of cecal tissue (*r*_s_ = 0.52, *p* = 0.0001, *n* = 48), the weight of cecal digesta (*r*_s_ = 0.34, *p* = 0.02, *n* = 48), and the pH in cecal digesta (*r*_s_ = 0.39, *p* = 0.006, *n* = 48) using Spearman’s rank correlation (Table 3). In contrast, the IgA flow rate of saliva per weight of submandibular gland tissue was not correlated with the concentration of IgA in cecal digesta (*r*_s_ = 0.15, *p* = 0.3, *n* = 48, Table 3).

### 3.4. Determination of Causal Effects Using Bayesian Network Analysis

**Figure 4:**
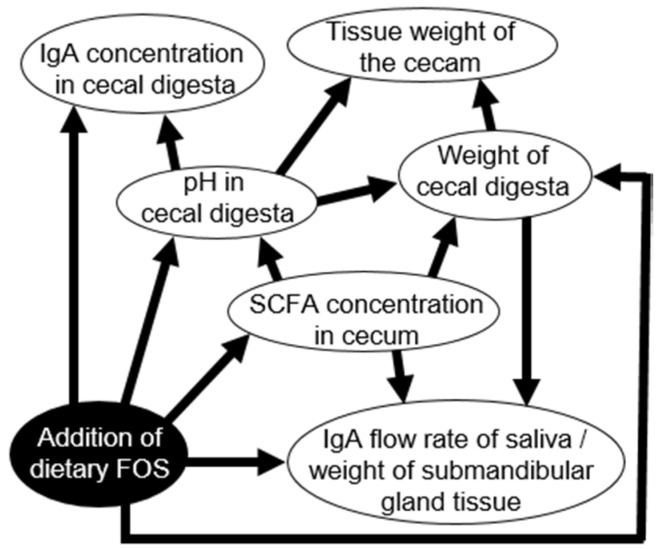
Causal effects between fructooligosaccharides (FOS) addition in diets, IgA flow rate of saliva per rat submandibular gland tissue weight, weight of cecal tissue, weight of cecal digesta, pH in cecal digesta, and IgA concentration of cecal digesta using Bayesian network analysis. Causes and effects are indicated by arrowheads and lines, respectively. The Bayesian network showed that the IgA flow rate of saliva per weight of submandibular gland tissue was affected by SCFA concentration in cecal digesta, weight of cecal digesta and addition of dietary FOS. On the other hand, the IgA concentration in cecal digesta and the IgA flow rate of saliva per weight of submandibular gland tissue were not causally related.

The Bayesian network showed that the IgA flow rate of saliva per weight of submandibular gland tissue was directly affected by the SCFA concentration in the cecal digesta, the weight of the cecal digesta, and addition of dietary FOS (Figure 4). In contrast, the IgA flow rate of saliva per weight of submandibular gland tissue had no direct relationship with IgA concentrations in the cecal digesta (Figure 4).

## 4. Discussion

The present study confirmed the effects of FOS ingestion on secretion of IgA in saliva and SCFA concentration in the large intestine in rats. Ingestion of FOS increased the IgA flow rate of saliva per weight of submandibular gland tissue at one and eight weeks (Figure 3C). 

### 4.1. Effect of SCFA in Cecal Digesta on IgA Flow Rate in Saliva

The Bayesian network analysis showed that the IgA flow rate of saliva per weight of submandibular gland tissue was directly affected by the SCFA concentration in cecal digesta (Figure 4). The SCFA concentration in cecal digesta of the FOS group was higher than that of the control group (Table 2). Accordingly, a higher IgA flow rate of saliva per weight of submandibular gland tissue should be induced by high concentration of SCFA with FOS ingestion.

Correlation between IgA flow rate of saliva per weight of submandibular gland tissue and SCFA concentration in cecal digesta showed extremely high (Table 3). This high correlation suggests that the concentration of SCFA in cecal digesta modulates the IgA flow rate of saliva per weight of submandibular gland tissue, which does not contradict the Bayesian network analysis (Figure 4).

### 4.2. Sympathetic Nerves and SCFA

Nervous system activity participates in metabolic homeostasis by detecting peripheral signaling molecules derived from dietary fiber intake [26]. SCFAs are signaling molecules that bind to the specific G-protein-coupled receptors free fatty acid receptor FFA3 (or GPR41) and FFA2 (or GPR43) and activate the sympathetic nervous system [27]. FFA2 and FFA3 exist in the sympathetic nervous system of rats, mice, and humans [28,29]. FFA3 is abundantly expressed in enteroendocrine cells and sympathetic neurons of the superior cervical ganglion [30]. SCFA has been shown to activate the sympathetic nervous system at enteroendocrine cells and/or sympathetic ganglia through FFA3 [28]. Actually, administration of propionate increased the heart rate in wild-type mice but not in GPR41^−/−^ mice [29], which suggested that the sympathetic nerve was stimulated by SCFA.

SCFA increases the epithelial cell mass and absorptive surface area, stimulating epithelial cell proliferation and causing the tissue weight to markedly increase in the small and large intestines [31,32]. However, when SCFA was introduced intraluminally into the colon, there was no increase in intestinal epithelial cells in sympathectomized rats [32]. Thus, SCFA activated sympathetic nerves.

### 4.3. Salivary IgA and Sympathetic Nerves

Carpenter et al. [33] reported that secretion of salivary IgA is upregulated by nerve impulses and that sympathetic nerves induce a greater effect than parasympathetic nerves in submandibular saliva in rats. Activation of the sympathetic-adrenal-medullary axis by pain stress induced higher salivary IgA concentrations in women with primary dysmenorrhea [34], which indicated that salivary IgA is controlled by the sympathetic nerves. SCFA stimulation of epithelial cell proliferation in the large intestine epithelium is also mediated by the sympathetic nervous system [35]. In the present study, the SCFA concentration in the cecal digesta of the FOS group was higher than that of the control group (Table 2). Higher SCFA levels in the large intestine with FOS intake would thus activate the autonomic nervous system, which could increase secretion of salivary IgA through sympathetic nerves.

### 4.4. Sympathetic Nerves and pIgR

The sympathetic nervous system dominates expression of polymeric immunoglobulin receptor (pIgR) in the submandibular glands [33]. Secretion of IgA into saliva depends on the expression of pIgR [33]. Our previous study showed higher expression of pIgR in the submandibular gland tissue and a higher flow rate IgA of saliva in rats fed diets supplemented with FOS and a mixture of indigestible carbohydrates [20]. In the present study, ingestion of FOS increased the IgA flow rate of saliva per weight of submandibular grand tissue (Figure 3C). pIgR could, therefore, be upregulated through sympathetic nerves with higher SCFA in the FOS group.

### 4.5. Difference in Mechanisms of Increased Salivary IgA Secretion and IgA in Cecal Digesta

The IgA flow rate of saliva per weight of submandibular gland tissue at one week was higher than that at week zero (Figure 3C), whereas the IgA concentration in cecal digesta at one week was not different from that at week zero in the FOS group (Figure 3C). Accordingly, the increase in salivary IgA secretion occurred earlier than that of cecal secretion in the FOS group. Furthermore, the Bayesian network analysis showed that the IgA flow rate of saliva per weight of submandibular gland tissue had no relationship with IgA concentrations in cecal digesta (Figure 4). This suggests that the mechanism of IgA increase in saliva might differ from that in cecal digesta.

There are two mechanisms for increased salivary IgA secretion: one is mediated by the nervous system, and the other is mediated by Peyer’s patch and nasopharynx-associated lymphoid tissue (NALT) [34,35,36]. Administration of an oral bacterial vaccine in humans increased the level of antigen-specific IgA^+^ B cells in saliva from the intestinal tract on day 50 [37]. This increase in salivary IgA secretion was mediated by Peyer’s patch and NALT [35,36]. On the other hand, pulse stimulation at 50 Hz (2-ms pulse width at 5 V) for 2 min on the sympathetic trunk in the sectioned rat neck increased the salivary IgA flow rate through the sympathetic nervous system in seconds [38], much faster than that mediated by Peyer’s patch and NALT [37,38]. As discussed above, the increased IgA flow rate in saliva with FOS intake observed herein could be mediated by the autonomic nervous system and thus occurred relatively early compared to expectations for mediation by Peyer’s patch and NALT [37,38]. Because the production rate of SCFA in the large intestine should be much slower than that of the autonomic nervous system reaction [39], the rate-limiting step for salivary IgA secretion would be the SCFA production rate in the large intestine. In this context, ingestion of fermentable fibers, such as FOS, could increase salivary IgA secretion as soon as the SCFA concentration increased in the large intestine, and it would remain high with continuous ingestion of fermentable fibers.

The concentration of IgA in cecal digesta is mediated by Peyer’s patch and gut-associated lymphoid tissue (GALT) [40]. Secretion of IgA in saliva could be controlled by the production rate of SCFA, as discussed above, whereas secretion of IgA in the cecum should be controlled by Peyer’s patch and GALT [40]. A previous study indicated that oligosaccharide intake increased the SCFA concentration, but did not increase the concentration of IgA in human feces [41]. Thus, the FOS-induced IgA level in the large intestine cannot be explained by the SCFA level in the large intestine and feces, indicating that the increase in salivary IgA level should occur through a mechanism distinct from that in the cecum. Consistent with this conclusion, we observed no direct relationship between IgA concentrations in cecal digesta and SCFA (Figure 4) and no correlation between the IgA flow rate of saliva per weight of submandibular gland tissue and the IgA concentration in cecal digesta.

### 4.6. Development of Secretion of IgA and Concentration of IgA in Saliva

The salivary IgA concentrations at four and eight weeks were higher than that at zero weeks in the control group (Figure 3B). The rats studied herein were 5–13 weeks old (see Materials and Methods), which corresponds to humans in early to late elementary school [42]. A previous study showed that younger elementary school students produced lower levels of salivary IgA [12], as did elderly populations [13,43]. Low levels of IgA in saliva are associated with a high incidence of upper respiratory tract infections (URTI) [7,9] and URTI recurrence [10]. Thus, elevating salivary IgA levels with fermentable fibers such as FOS could be quite important for young populations to prevent URTI.

The flow rate of saliva and weight of submandibular gland tissue increased with experiment duration (Figure 1), indicating submandibular gland development in the rats between five and 13 weeks, and the flow rate of saliva also increased with submandibular gland development. Furthermore, the IgA flow rates in saliva per weight of submandibular gland tissue at four and eight weeks were higher than that at zero weeks in the control group (Figure 3C), indicating concomitant development of IgA production per unit of submandibular gland volume.

### 4.7. Estimation of the Impact of Lactate in the Cecal Digesta

Lactate was detected in the cecal digesta of the FOS group at eight weeks in this study (Table 2). In a previous study, the number of lactate-producing bacteria in the cecal digesta at two weeks was higher than that at zero weeks in the FOS group [44]. However, this increase in the number of the lactate-producing bacteria with FOS addition disappeared at eight weeks [44]. Thus, the number of the lactate-producing bacteria in the cecal digesta at eight weeks would have been the same as that at zero weeks in the FOS group in this study. Hence, the lactate-producing bacteria at eight weeks would not have been associated with the higher salivary IgA secretion upon FOS addition in this study. Accordingly, the higher salivary IgA secretion with FOS addition in the present study was likely not mediated by effects of Peyer’s patch and NALT with the lactate-producing bacteria.

In our previous study, a diet with addition of polydextrose and lactitol, which are kinds of indigestible carbohydrates, resulted in higher salivary IgA secretion and no lactate in the cecal digesta at eight weeks in rats (unpublished data, Yamamoto et al.). Thus, salivary IgA secretion would not be associated with the lactate concentration in the cecal digesta. Therefore, the existence of lactate and the number of lactate-producing bacteria in the cecum might not induce salivary IgA secretion.

### 4.8. Considerations

We established the time after feeding as an independent variable for two-way layout in this study. However, we cannot rule out the existence of a confounding factor in the time after feeding. The rats employed in this study showed increased body and tissue weight, and we cannot distinguish the effects of the time after feeding from those of development of rats. We suggested the possibility that ingestion of FOS for 1–8 weeks would increase secretion of salivary IgA even in humans. Although it is possible that the increase in body and submandibular gland tissue might affect the duration of FOS feeding time, our results strongly suggest that ingestion of FOS increased salivary IgA secretion by high levels of SCFA in the large intestine. Since ingestion of FOS increases SCFA levels in the large intestine at short (e.g., 12 h) and long times (e.g., 27 weeks) [44,45], continuous ingestion of FOS is expected to increase IgA levels in the large intestine of humans.

It has been common to add more than 50 g/kg FOS in diets in the animal experiments [15,46]. On the other hand, the tolerance level of FOS is 0.3 g/kg of body weight for normal Japanese people [47]; therefore, determination of the reasonable level of FOS intake to increase salivary IgA levels requires additional research.

## 5. Conclusions

In conclusion, ingestion of FOS increased salivary IgA secretion by stimulating the high levels of SCFA which was produced with fermentation in the large intestine. Our results suggest the possibility that the increase in salivary IgA occurs through a different mechanism from that in the cecum, which is mediated by Peyer’s patch and GALT.

## Figures and Tables

**Table 1:** The composition of control and fructooligosaccharides (FOS) diets (g/kg).

Ingredient	Control	FOS
Casein	200	200
Sucrose	700	650
Corn oil	50	50
Mineral mixture (AIN-76) *	35	35
Vitamin mixture (AIN-76) ^†^	10	10
dl-Methionine	3	3
Choline bitartrate	2	2
FOS		50
Total	1000	1000

* Mineral mixture (AIN-76) (g/kg): calcium phosphate 500, sodium chloride 74, potassium citrate 220, potassium sulfate 52, magnesium oxide 24, magnesium carbonate 3.5, ferric citrate 6, zinc carbonate 1.6, cupric carbonate 0.3, potassium iodate 0.01, sodium selenite 0.01, chromium potassium sulfate 0.55, sucrose 118. ^†^ Vitamin mixture (AIN-76) (mg/kg): thiamin 600, riboflavin 600, pyridoxin 700, niacin 300, calcium pantothenate 160, folic acid 200, biotin 200, cyanocobalamin 10, retinol 24,000, cholecalciferol 2.5, tocotrienols 5000, menadione 5, and sucrose 979,000.

**Table 2:** Concentration of organic acids in cecal contents at eight weeks after feeding (mmol/kg digesta).

Acids	Control		FOS ^$^		*p* ^†^
	Mean	SE ^$$^	Mean	SE	
Acetate	17.4	0.2	25.6	1.9	0.1
Propionate	7.06	0.24	7.58	0.41	0.7
*n*-Butyrate	3.58	0.25	15.21	1.00	0.004
Lactate	N.D. ^‡^	-	6.56	1.05	-
SCFA ^§^	33.4	0.2	48.5	2.3	<0.05

*n* = 6; **^$^** FOS: fructooligosaccharides; ^†^ calculated using Welch’s *t*-test. **^$$^** SE: Standard error; ^‡^ N.D.: lactate in the cecal digesta were not detected in the control group. ^§^ SCFA (short-chain fatty acids): the sum of acetate, propionate, and *n*-butyrate.

**Table 3:** Correlation between IgA flow rate of saliva per weight of submandibular gland tissue and concentration of short-chain fatty acids (SCFA) in cecal digesta, weight of cecal tissue, weight of cecal digesta, pH in cecal digesta, or concentration of IgA in cecal digesta.

Variable	IgA Flow Rate of Saliva per Weight of Submandibular Gland Tissue		
	*r_s_* ^$^	*p*	*n*
SCFA concentration in cecal digesta	0.86	0.0006	12
Weight of cecal tissue	0.52	0.0001	48
Weight of cecal digesta	0.34	0.02	48
pH in cecal digesta	0.39	0.006	48
Concentration of IgA in cecal digesta	0.15	0.3	48

SCFA: short-chain fatty acids, which was the sum of concentration of acetate, propionate, and *n*-butyrate. **^$^** Spearman’s rank correlation coefficient. The concentration of SCFA was measured only at eight weeks, so only 12 samples were included. For all other parameters, 48 individuals were included.
